# Fungal Otitis Externa (Otomycosis) Associated with *Aspergillus Flavus*: A Case Image

**DOI:** 10.1007/s12105-023-01606-1

**Published:** 2024-02-09

**Authors:** William W. MacDonald, Paul E. Wakely, John R. Kalmar, Prokopios P. Argyris

**Affiliations:** 1 Department of Pathology, The Ohio State University Wexner Medical Center, James Cancer Hospital and Solove Research Institute, Columbus, OH USA; 2 Division of Oral and Maxillofacial Pathology, The Ohio State University College of Dentistry, Postle Hall, Room 2191 305 W. 12th Ave, Columbus, OH USA

**Keywords:** Aspergillus, Otomycosis, Fungal otitis externa, External auditory canal, Ear, Cholesteatoma

## Abstract

A 48-year-old man presented with a chief complaint of intermittent right ear otorrhea of several-month duration, occasional otalgia and progressive unilateral hearing impairment. He also reported frequent episodes of headache and pressure in the sinuses and maxilla. Previous systemic treatment with antibiotics failed to alleviate the symptoms. A head/neck CT showed completely normal mastoid, middle ear and external auditory canal regions without any evidence of opacification or bone erosion. Otoscopic examination of the right ear disclosed aggregates of dried, brown, fibrillar material and debris occluding the external auditory canal and obstructing the otherwise intact tympanic membrane. Dilation of the external auditory canal or thickening of the tympanic membrane were not appreciated. The canal was debrided and the fibrillar material was placed in formalin. Histopathologic examination revealed numerous branching, septated fungal hyphae organized in densely-packed clusters. In other areas, the fungal hyphae abutted or were attached to lamellated collections of orthokeratin. As highlighted by GMS staining, the fungi were morphologically compatible with *Aspergillus* species. The clinicopathologic findings supported a diagnosis of fungal otitis externa, while the numerous anucleate squamous cells were compatible with colonization of an underlying, probably developing, cholesteatoma. Culture of material isolated from the external auditory canal confirmed the presence of *Aspergillus flavus.* In this illustrative case, we present the main clinical and microscopic characteristics of Aspergillus-related otomycosis developing in the setting of a tautochronous cholesteatoma.

A 48-year-old man presented to the ENT clinic with a chief complaint of intermittent right ear otorrhea of several-month duration, occasional otalgia and progressive unilateral hearing impairment. He also reported frequent episodes of headache and a feeling of pressure in the sinuses and maxilla. The patient’s medical history was otherwise unremarkable and a systemic course of antibiotics failed to alleviate his symptoms. A head/neck CT showed completely normal mastoid, middle ear and external auditory canal regions without any evidence of opacification or bone erosion. Otoscopic examination of the right ear disclosed aggregates of dried, brown, fibrillar material occluding the external auditory canal and obstructing the tympanic membrane which remained intact. Dilation of the external auditory canal or thickening of the tympanic membrane were not appreciated. Debridement of the fibrillar material was performed. Histopathologic examination revealed numerous branching septate fungal hyphae organized in densely-packed clusters (Fig. 1A). In other areas, fungal hyphae abutted or were attached to lamellated collections of orthokeratin (Fig. 1B). Under high-power magnification, the fungi were morphologically compatible with *Aspergillus spp.* (Fig. 1C). Grocott-Gomori methenamine silver (GMS) stain highlighted the hyphal walls of the microorganisms (Fig. 1D). The clinical and microscopic findings were diagnostic of fungal otitis externa, while the numerous anucleate squamous cells were compatible with an underlying, probably developing, cholesteatoma. Culture of material isolated from the external auditory canal confirmed the presence of *Aspergillus flavus.*

**Fig. 1:**
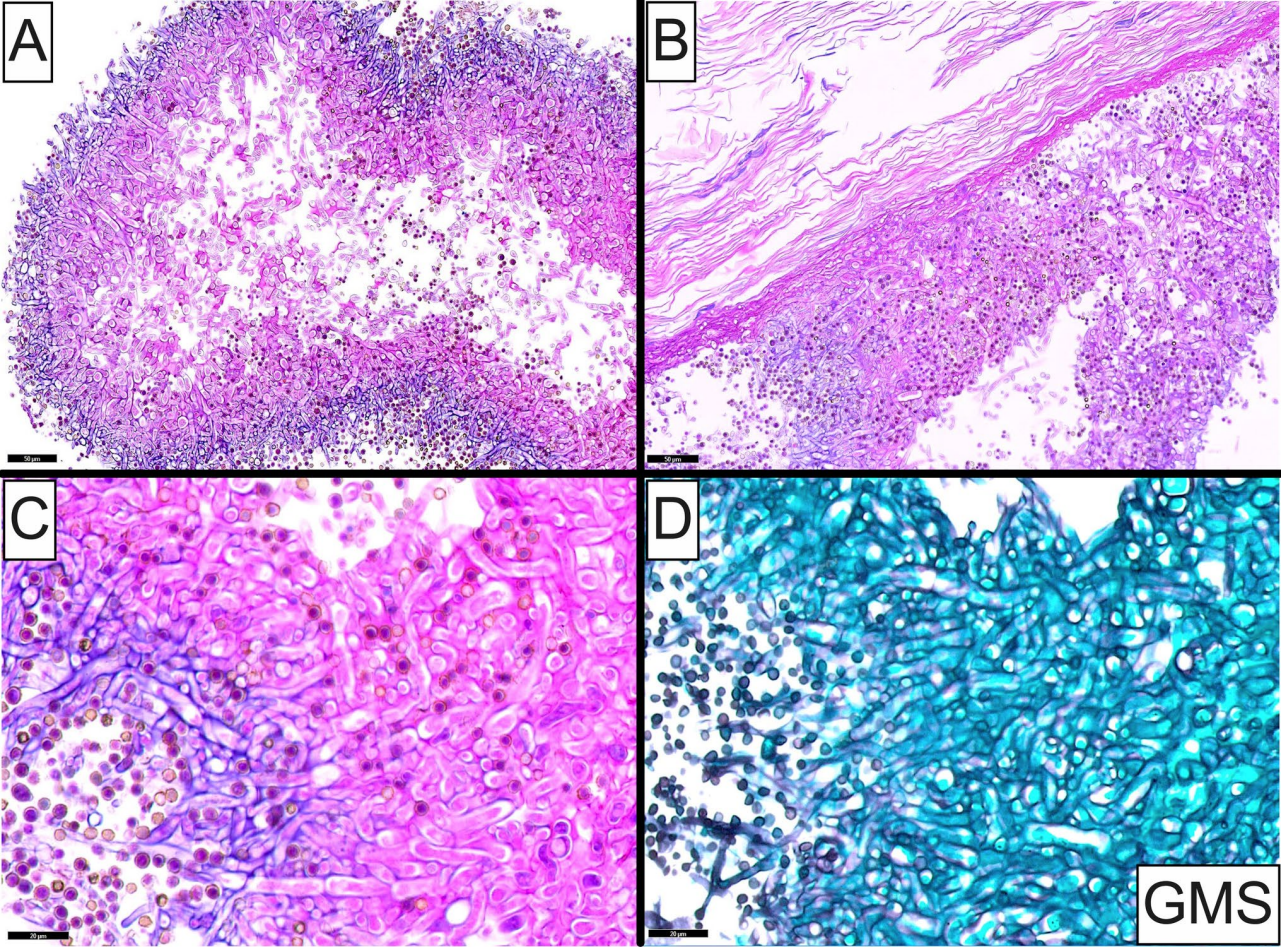
**A** Medium-power photomicrograph depicting numerous branching septate fungal hyphae organized in a densely-packed clusters. **B** Medium-power photomicrograph showing the fungal microorganisms abutting or attached to lamellated anucleate squamous cells, compatible with cholesteatoma. **C** High-power photomicrograph showing branching, septated fungi morphologically compatible with *Aspergillus* species. **D** Grocott-Gomori methenamine silver (GMS) stain highlighting the hyphal walls of the microorganisms.

*Aspergillus spp.*, including *A. fumigatus*, *A. flavus* and *A. niger*, represent ubiquitous, environmental, filamentous, fungal microorganisms generally acquired through inhalation of airborne spores. Fungal infections of the head/neck associated with *Aspergillus spp.* chiefly involve the paranasal sinuses and exhibit markedly diverse clinical presentations ranging from life-threatening invasive fungal sinusitis to innocuous mycetomas [1]. Otitis externa of fungal etiology, also known as otomycosis, comprises approximately 9% of all otitis externa cases with the remaining cases associated with bacterial infections. *Aspergillus* and *Candida spp*. represent the most common causative agents [1, 2], while other species including *Mucor*, *Fusarium*, *Scedosporium* and *Cryptococcus* have been rarely reported. Among the *Aspergillus* family, *A. niger* is most commonly associated with otomycoses, whereas *A. fumigatus* and *A. flavus*, as seen in this case, are less frequent [3].

The symptomatology of fungal otitis externa is nonspecific and includes otalgia, long-term relapsing otorrhea, hearing loss, aural fullness and pruritus. In immunocompetent individuals, fungal colonization is unilateral, superficial, and usually confined to the external auditory meatus [1, 2, 4]. Rare complications such as serous otitis media and tympanic membrane perforation are seen in roughly 14% of otomycosis patients [4]. Various predisposing factors have been identified including humid climate, inadequate hygiene, topical or systemic immunosuppression, history of ear surgery and prolonged use of ototopical antibiotics [1]. In immunosuppression, otomycosis tends to present bilaterally [3] with acute or chronic invasive fungal infections developing in the middle and inner ear [2]. An association between otomycosis and tautochronous cholesteatoma, as indicated based on histopathology in the current patient, has been previously documented [2, 5]. Histopathologic examination and/or culture for the detection of fungal microorganisms, in conjunction with otoscopic evaluation, are required for proper diagnosis and management of otomycosis. Mechanical debridement along with topical antifungal treatment are considered curative [1, 2, 4].

## Data Availability

All available clinical and histopathologic data pertaining to the current case report are presented in the manuscript.
